# Characterization of Benchtop-Fabricated Arrays of Nanowrinkled Surface Electrodes as a Nitric Oxide Electrochemical Sensor

**DOI:** 10.3390/bios13080794

**Published:** 2023-08-07

**Authors:** Cindy Peto-Gutiérrez, Genaro Vázquez-Victorio, Mathieu Hautefeuille

**Affiliations:** 1 Laboratorio Nacional de Soluciones Biomiméticas para Diagnóstico y Terapia (LaNSBioDyT), Universidad Nacional Autónoma de México, Mexico City 04510, Mexico; 2 Departamento de Física, Facultad de Ciencias, Universidad Nacional Autónoma de México, Mexico City 04510, Mexico; 3 Laboratoire de Biologie du Développement (UMR 7622), Institut de Biologie Paris Seine, Sorbonne Université, 75005 Paris, France

**Keywords:** nanowrinkled surface electrodes, extracellular media sensing, nitric oxide

## Abstract

In this work, we present an accessible benchtop fabrication technique to obtain a planar array of gold nanowrinkled surface electrodes (ANSE) for the construction of electrochemical cells, specifically to monitor soluble biomarkers of interest in cell culture environments. We present a complete characterization of the array and its response as an electrochemical cell. To validate our sensor, we evaluated the device sensitivity to detect nitric oxide (NO), an important molecule produced by endothelial cells as a response to environmental signals such as mechanics and growth factors. While testing measurements of nitric oxide in aqueous solutions with isotonic salt concentrations, we evidenced the influence of the environmental conditions for such electrochemical measurements, showing that the aqueous medium, usually not accounted for, significantly impacts the outcome. Finally, we present the application of the electrochemical sensor for the detection of nitric oxide released from stimulated endothelial cells as a proof of concept.

## 1. Introduction

Electrochemical sensors have always had an important role in biomarker monitoring because of their remarkable sensitivity, selectivity, and stability, making them a popular choice for a wide range of applications, including environmental, healthcare, and industrial process control [1,2,3]. A lesser known area of application is the analysis of extracellular media in typical in vitro platforms or even within the more sophisticated microfluidic chips adapted for biomimetic studies [4,5]. Additionally, electrochemical sensors present themselves as elements within lab-on-a-chip (LoC) platforms and point-of-care diagnostics (PoC) [6,7]. All of these applications require the integration of microelectrodes, typically in a planar arrangement as transduction elements for on-chip electrochemical sensing. Various methods to fabricate arrays of microelectrodes have been reported to date, such as photolithography techniques [8,9]. Despite the robustness and reproducibility of these techniques, they are typically time-consuming and expensive due to the need for clean-room facilities. Alternative benchtop fabrication methods like doctor-blading, adhesive film mask fabrication by xurography, ink-jet printing, and soft lithography overcome these limitations and provide rapid and on-demand prototyping [10,11,12].

To design electrochemical sensors, the properties of the electrode surface must also be considered, as these properties determine the efficiency of the electron transfer reaction between the analyte and the electrode. The use of electrodes with nanostructured surfaces is a promising approach to improve the performance of electrochemical sensors by increasing the available surface area where the analyte is adsorbed, thus leading to more electron transfer events and therefore to higher sensitivity. There are currently several methods to add nanoparticles to the electrode surface [13,14], but there are only a few reports on the intrinsic nanostructuring of the conductive material [15,16]. The fabrication of gold electrodes with nanostructured surfaces via the thermally induced shrinking of a prestressed polystyrene sheet was reported previously, and in combination with commercial counter and reference electrodes, the electrochemical sensing capabilities of these benchtop-fabricated electrodes were demonstrated [17,18].

In this work, we extended the mentioned fabrication technique to obtain planar arrays of gold nanowrinkled-surface electrodes (ANSE) with the aim of using these as electrochemical cells, specifically to monitor soluble biomarkers of interest in biological environments. We present a complete characterization of the behavior of the array as an electrochemical cell while questioning the influence of the conditions necessary for it to monitor biological environments in situ. Furthermore, we evaluate the device sensitivity to detect nitric oxide (NO), which is an important cell mechano-signaling molecule. Finally, we present the application of the electrochemical sensor to the detection of nitric oxide released from stimulated endothelial cells as a proof of concept.

## 2. Materials and Methods

### 2.1. Materials and Reagents

Sodium nitrite (NaNO_2_), ferrous sulfate (FeSO_4_), sulfuric acid (H_2_SO_4_), potassium ferricyanide (K_3_Fe(CN)_6_), potassium ferrocyanide (K_4_Fe(CN)_6_), a nitrite assay kit (Griess reagent, MAK367), and Dulbecco’s phosphate-buffered saline (DPBS 10X) were received from Sigma Aldrich, Merck. Gold target (diameter: 57 mm, thickness: 0.1 mm) with a purity of 99.99% was purchased from Agar Scientific, Essex, UK. Hank’s balanced salt solution (HBSS 10X), Dulbecco’s modified eagle medium mixture powder (DMEM), antibiotic-antimycotic (Anti-Anti 100X), and fetal bovine serum (FBS) were purchased from Gibco, Thermo Fisher Scientific, Waltham, MA, USA. Diaminofluorescein probe DAF-FM diacetate (D23844) was acquired from Invitrogen, Thermo Fisher Scientific.

### 2.2. Fabrication of Arrays of Nanowrinkled-Surface Electrodes

The electrochemical sensors fabricated fully in house consisted of a planar array of three 500 µm wide gold microelectrodes with nanowrinkled surfaces on a plastic polystyrene (PS) substrate with a size of 30 × 20 mm. The sensors were integrated into a PDMS well or microfluidic channel via chemical bonding, in order to define the reaction area and to contain the liquid samples.

**Figure 1:**
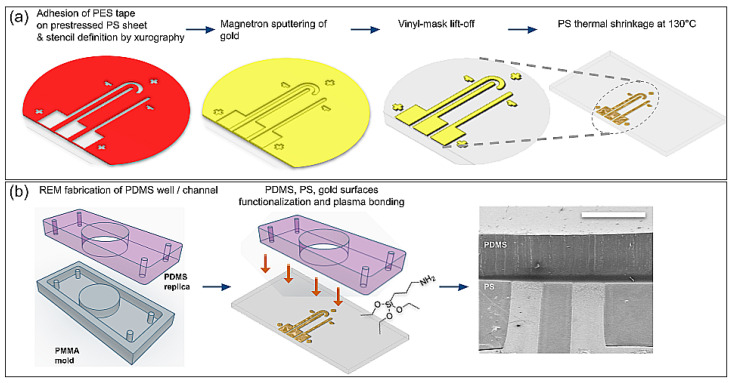
Fabrication process of the planar array of nanostructured surface electrodes (ANSE). (**a**) Benchtop lift-off process to define electrode shapes on top of a prestressed polystyrene sheet and subsequent temperature-induced shrinking, which leads to the formation of nanowrinkles at the surface of gold electrodes. (**b**) Steps to integrate an ANSE with a PDMS mask or microfluidic channel and photograph of the resulting interface as observed with SEM (scale bar = 1 mm).

Their fabrication, detailed here and summarized in the diagrams of Figure 1a, was based on the temperature-induced shrinking of the polystyrene substrate. Manufacturing was accomplished by using only benchtop techniques and instruments. The first step consisted of sticking a polyester tape with silicone adhesive (PES, Polyester Tape 8991, 3M, Saint Paul, MN, USA) onto a clean, non-scratched, prestressed polystyrene sheet (“Shrinky Dinks”, Just Play, Boca Raton, FL, USA), taking care that no air bubbles or dust was trapped between the tape and the polystyrene sheet. Next, a mask was cut onto the adhered tape using a crafts cutting plotter (CAMEO 3, Silhouette America, Lehi, UT, USA) following a 2D CAD design transferred with precision to the mask. The resulting mask presented the desired final shape of the three electrodes but with dimensions 2.5 times greater, i.e., the track defined by the adhesive mask had a width of 1.25 mm, so that after the shrinkage it ended up as a 500 µm wide electrode. A thin gold layer of 20–40 nm was then deposited onto the masked substrate via magnetron sputtering (SC7620 Mini Sputter Coater, Quorum Technologies, Lewes, UK), using a plasma current of 18 mA for 180 s. Next, the adhesive mask was carefully lifted off to leave the desired electrode pattern on the PS sheet. Finally, this substrate with the transferred gold tracks was placed inside an oven at 130 °C, on top of a preheated, flat plate of aluminum to avoid inhomogeneous shrinkage and to promote flatness of the final sample. Immediate shrinking was observed, but the process was left to stabilize for 5 min. To perfectly flatten the plastic substrate, it was then transferred to a preheated glass substrate and the oven temperature was raised to 150 °C. This shrinking process of the PS sheet induced isotropic wrinkling of the thin metal layer deposited on it, and it improved the adhesion of gold to the plastic substrate, thus leaving three conductive tracks of 500 µm width, hereby called electrodes. This nanostructuring principle was kindly shared to us by José Moran-Mirabal and his team [19].

The PDMS channel or well to be integrated with the ANSE was obtained via the soft lithography technique of replica molding (REM) from an acrylic (PMMA) mold manufactured by simple CNC micromachining (Figure 1b). The PDMS surface was air-plasma cleaned for 1 min at the highest RF power (PE25 series plasma system, Plasma Etch, Carson City, NV, USA) and immediately submerged in a freshly prepared solution of 5% APTES in 70% ethanol; 2 h later, the PDMS surface was gently rinsed with 70% ethanol and CDA-dried. Finally, both the functionalized PDMS surface and the Au/PS surface were exposed to air plasma for 3 min and promptly attached.

### 2.3. Geometric and Topographical Characterizations

Scanning electron microscopy (JCM-6000 PLUS “Neoscope”, JEOL, Peabody, MA, USA) was used to observe the nanometric structures of the electrode surface. The geometric dimensions before and after shrinking were measured on the pictures taken with a stereoscopic microscope (SMZ745T, Nikon, Tokyo, Japan).

### 2.4. Electrochemical Characterizations

**Figure 2:**
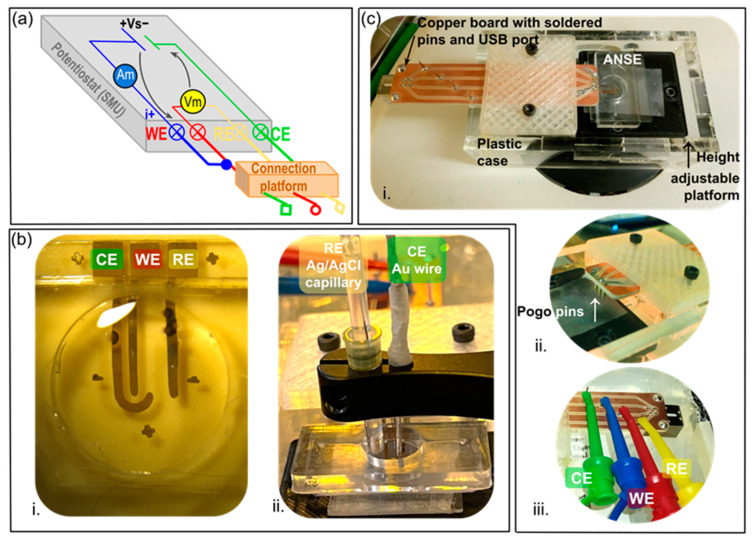
Instrumentation for electrochemical characterizations. (**a**) Potentiostat connections to an electrochemical cell in a three-electrode configuration; Vs = voltage source, Vm = voltmeter, Am = ammeter, WE = working electrode, RE = reference electrode, CE = counter electrode. (**b**) Electrode configuration for (i) electrochemical sensing with the ANSE, or (ii) characterization of the electrochemically active surface of each nanowrinkled surface electrode (analyzed electrode is plugged as the WE). (**c**) Ad hoc connection platform based on spring-loaded pins (pogo pins); (i) image of the complete platform, (ii) detail of pogo pins spaced equally as the contact pads of the array, (iii) detail of the in-house fabricated copper board with soldered pins which allow for versatile wire connections.

The cyclic voltammetry and chronoamperometry techniques described hereafter were performed with a Source Meter Unit (SMU 2450, Keithley Instruments, Cleveland, OH, USA), upgraded to function as a potentiostat (EC Upgrade scripts, Keithley Instruments, Cleveland, OH USA) in a three-electrode configuration, as schematized in Figure 2a. To ensure long-term, robust, and stable connections between the SMU front terminals and the electrodes in the array during measurement instrumentation, a platform was custom-built in house, based on spring-loaded (pogo) pins spaced equally and placed upon the contact pads designed especially on the electrodes (Figure 2c).

#### 2.4.1. Characterization of Electrochemically Active Surface Area

Each electrode in the array was characterized to obtain a measurement of its electrochemically active surface area (EASA). The geometric area to be analyzed was defined with a 11 mm diameter PDMS well, as observed in Figure 2b. The analyzed electrode, in turn, was connected to the pogo-pin platform as the working electrode (WE) of a cell, containing also a reference silver/silver chloride electrode (RE) and a gold wire as a counter electrode (CE). Cyclic voltammetry sweeps between 0 and 1.5 V were performed with the electrodes in contact with a 100 mM H_2_SO_4_ solution at a scan rate of 100 mV/s. This yielded an anodic current peak corresponding to the oxidation of the gold layer, other oxidation phenomena, and a subsequent cathodic current peak due to the reduction of the gold oxide layer. The cathodic peak of the last scan was integrated to obtain the transferred charge for the gold oxide layer reduction (µC). The EASA (cm^2^) was then calculated from the theoretical surface charge density of a gold oxide monolayer, namely 386 µC·cm^−2^, using the equation EASA = transferred charge/surface charge density [20].

#### 2.4.2. Evaluation of ANSE’s Electrochemical Performance

To evaluate the device’s ability to conduct voltammetric techniques in an aqueous solution, the electrodes in the array were connected to the corresponding terminals as indicated in Figure 2c. A solution of 1 mM ferrocyanide/ferricyanide (K_4_[Fe(CN)_6_]/K_3_[Fe(CN)_6_]) in 0.5 M KCl was dropped over electrodes that were contained in the integrated PDMS well. Cyclic voltammetry sweeps were implemented from −0.6 to 0.6 V at a scan speed of 100 mV/s. To evaluate the electrochemical system’s compliance with the Randles–Sevcik model [21], the same voltammetric experiment was repeated but at different scan speeds, 10, 30, 50, 75, 100, and 125 mV/s, and a higher ferrocyanide/ferricyanide concentration of 1 mM. The maximum current at the oxidation (positive current) and reduction (negative current) peaks was found and plotted against the square root of the scan speed.

To study the performance of the ANSE as an electrochemical sensor, chronoamperometric measurements (potential step of 0.5 V vs. pseudoreference electrode, for 5 s) were carried out in ferrocyanide solutions spanning the range 0.1 to 1000 µM, with KCl 0.5 M as the supporting electrolyte. To obtain a calibration curve, the maximum current (peak current) and the area under the curve in a 1 s interval starting at the maximum point (transferred charge) were extracted from each peak and plotted against the ferrocyanide concentration. The same experiment was repeated using a commercial silver/silver chloride reference electrode (Ag/AgCl RRPEAGCL, Pine Research, Durham, NC, USA) instead of the gold pseudoreference electrode. Also, a commercial array of screen-printed gold electrodes was challenged under the same technique to compare the capabilities of our low-cost nanowrinkled surface arrays of electrodes with those of commercially available electrodes.

### 2.5. Electrochemical Detection of Dissolved Nitric Oxide with an ANSE

#### 2.5.1. Preparation of Nitric Oxide Aqueous Solutions for Calibration

First, a 0.5 M KCl solution was prepared with deionized water and contained in a vial capped with a septum; then, the dissolved oxygen was displaced by bubbling nitrogen gas. Then, nitric oxide gas was produced from the anaerobic reaction between sodium nitrite (NaNO_2_) and an acid ferrous sulfate solution (FeSO_4_, H_2_SO_4_), using the syringes method reported by Bruce Mattson [22]. The gas contained in the syringe was bubbled in the deoxygenated solution of KCl 0.5 M. The concentration of nitric oxide in the saturated solution was measured via the absorbance Griess assay, and dilutions were prepared while avoiding contact with oxygen.

#### 2.5.2. NO Electrooxidation Calibration Curve

For the calibration curve experiments, a stock solution of nitric oxide was prepared by bubbling nitric oxide gas into a 0.5 M KCl deoxygenated solution. A 1:20 dilution was prepared in the same supporting electrolyte, and its nitric oxide concentration was measured with the help of the colorimetric Griess assay. Small volume aliquots were subsequently added to 100 µL of the supporting electrolyte (KCl 0.5 M) to increase the NO final concentration. NO electrooxidation was initiated after each addition by applying a 0.65 V potential step, and the anodic current was recorded (chronoamperometry). The peak area in a 1 s window was integrated to calculate the transferred charge, and this was plotted against the nitric oxide concentration in the solution after the aliquot addition.

### 2.6. Detection of Nitric Oxide Released from Stimulated Endothelial Cells in Culture

#### 2.6.1. Cell Culture Conditions

The immortalized human liver endothelial cell line TMNK-1 (RRID:CVCL_4W79, kindly provided by Marina Macias-Silva) was used as an endothelial cell model and maintained in high glucose DMEM complemented with 10% fetal bovine serum (FBS) and 1% Anti-Anti (Gibco, Thermo Fisher Scientific). For the experimental conditions, the TMNK-1 cells were cultured in fibronectin-coated polystyrene wells of a 48-well plate, using DMEM supplemented with 1% Anti-Anti. After 24 h of culture, having a confluent monolayer, two different assays were carried out for stimulation with vascular endothelial growth factor (VEGF) and then for quantification of the production of nitric oxide.

#### 2.6.2. Nitric Oxide Colorimetric Detection

Nitrite, an oxidation product of nitric oxide, was detected in the extracellular media using the Griess assay. The culture medium was removed from the wells, and then, the wells were rinsed twice with HBSS 1×. A total of 130 µL of a solution containing 50 ng/mL of VEGF in HBSS 1× or DPBS 1× was then added to each well. This concentration of VEGF is known to rapidly stimulate the synthesis of NO by endothelial cells and especially liver endothelial cells [23]. As a negative control, the cells were also incubated in the background solution with no stimulant. Ten minutes past incubation at 37 °C, 100 µL of each extracellular solution was transferred to a 96-well plate. Immediately afterwards, Griess reagents I and II, and an assay buffer were added to each well containing a sample or background solution, as indicated in the manufacturer’s protocol. The plate was assayed for UV-Vis absorbance at 540 nm in a BioTek Synergy H1 Plate Reader at 20 °C. The nitrite concentration in each well was calculated from the proper calibration curve. The culture well was “refilled” with DMEM containing 1 µM calcein AM and 5 µM propidium iodide (PI) to evaluate the viability of cells after the stimulation assay.

#### 2.6.3. Detection of Nitric Oxide with Fluorescent DAF-FM Diacetate

Cells were serum-starved for overnight (12–16 h) prior to the stimulation. Afterwards, serum-free media were replaced by either of the following three options: (1) DMEM supplemented with 50 ng/mL of VEGF, (2) DMEM with 0% FBS, or 3) DMEM with 10% FBS. After two hours of stimulation, the media were rinsed with HBSS 1×, and a solution containing 10 µM 4-amino-5-methylamino-2′,7′-difluorofluorescein (DAF-FM) diacetate and 5 µM propidium iodide (PI) in HBSS 1× was added. Fluorescent images of each assay well were taken (ZOE Fluorescent cell imager, BIO-RAD, Hercules, CA, USA) at approximately 10, 30, and 60 min after the addition of DAF-FM at the same light intensity conditions. Using ImageJ (ImageJ, U. S. National Institutes of Health, Bethesda, MD, USA), we converted the green channel to grayscale, and the brightness conditions were set and propagated to all photographs (Figure S7). In every micrograph, we measured the fluorescence intensity as the average gray level. Finally, we computed the average and standard deviation across all micrographs corresponding to the same stimulation and capture time conditions.

#### 2.6.4. Detection of Nitric Oxide with the ANSE-Based Electrochemical Sensor

A confluent TMNK cell monolayer was stimulated with 50 ng/mL of VEGF dissolved in HBSS 1× or in DPBS 1× following the same procedure as that for the colorimetric detection. After 10 min of stimulation, 100 µL of the solution was transferred to the PDMS well with integrated ANSE in which a concentrated solution of the supporting electrolyte KCl was previously contained. A 0.65 V potential step was applied, and the oxidation current was recorded. The peak was integrated in a 1 s window to obtain the transferred charge (Q_tr_). The same chronoamperometric experiment was repeated three times. The response to the background solutions (DPBS and HBSS 1×) was also recorded with the same parameters.

## 3. Results

### 3.1. Fabrication and Characterization of Nanowrinkled-Surface Electrodes

**Figure 3:**
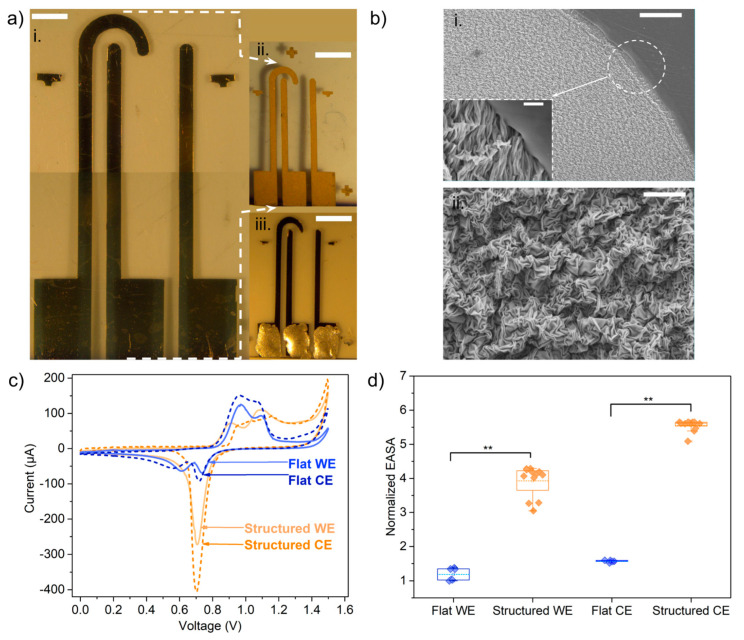
Arrays of nanostructured-surface electrodes and characterization of surface by SEM and cyclic voltammetry. (**a**) Picture of the device before (i) and after (ii) temperature-induced shrinking (ANSE), and flat-electrode array with the same geometric dimensions as the ANSE (iii); scale bars = 3 mm. (**b**) Scanning electron microscopy images of gold surface after shrinking showing the wrinkle-like nanostructures; scale bars at different magnifications: (i) 100 µm, (ii) 10 µm, inset = 5 µm. (**c**) Voltammograms obtained with the different indicated electrode types for the oxidation and reduction of gold in the acidic H_2_SO_4_ 0.1 M solution, the arrows point the cathodic peak appearing in the reverse scan portion, from which the area of the oxidation peak is extracted. (**d**) Comparison of electrochemically active surface area between flat-surface and nanostructured-surface electrodes; statistical significance: **—at *p* < 0.01, (n > 3).

The benchtop fabrication method described earlier was used to obtain arrays of electrodes with gold nanowrinkled surfaces on top of polystyrene thermoplastic substrates Figure 3ai. The arrays consisted of a straight working electrode, a straight pseudoreference electrode, and a longer auxiliary electrode with a hook-shaped end, as depicted in Figure 3bi. Despite being just a few nanometers thick, the electrodes after shrinkage presented a longitudinal resistance lower than 30 Ω, which was considered enough to conduct the faradaic currents of electrochemical processes. Characteristic geometric dimensions of the fabricated electrodes were measured from images taken with the scanning electron microscope (SEM) or a stereo microscope and were compared with the measurements of an electrochemically active surface area in order to assess the consistency of the manufacturing method and to gain insight regarding the sensitivity towards electrochemical measurements.

The width and geometric area of the electrodes were measured from transillumination images of the electrode arrays before and after shrinkage (Figure S1c), and the linear and area shrinking ratios were calculated. A ratio of 0.4 ± 0.0003 was calculated for width. In the case of the geometric areas, the calculated quotient was 0.179 ± 0.006. This is close to 0.16, which is stated by the manufacturer of the PS sheet as the “approximate area reduction”. The final width of the tracks after shrinking, as measured from the images, was 0.536 ± 0.036 mm, which corresponds to a discrepancy of only 7.2% with respect to the designed width.

As observed from SEM images (Figure 3b), the gold surface is structured in the form of nanowrinkles, as described in [16,17]. The width of the nanowrinkles was measured from SEM images; the average was found to be 0.425 ± 0.072 nm (Figure S1a). The polystyrene surface remained smooth after shrinking, which is useful for the later adhesion of the PDMS well or microfluidic channel.

In order to validate that the nanostructuring process would enhance the sensitivity per unit geometric area, the electrochemically active surface area (EASA) was estimated from the voltammogram cathodic peak integration (Figure 3c), and it was compared with that obtained for a flat-surface electrode with the same corresponding geometry. All EASA values (cm^2^) for each electrode type were normalized against the smallest surface area, which was that of the flat working (straight) electrode. The normalized EASA values are shown in Figure 3d.

It has been reported that during the shrinking process, the prestressed polystyrene sheet used in this work shrinks to 16% of its original area [24]. In this sense, the lateral dimensions of the electrode tracks before shrinking were designed to be 2.5 times larger than the final desired dimensions, which are the same as the electrodes that were kept flat. So, assuming isotropic shrinkage, 6.25 is the expected ratio of the areas of structured electrodes against flat electrodes having the same geometrical dimensions. In the case of straight electrodes, those with a structured surface exhibited an EASA only 3.93 (±0.45) times larger than the EASA of those with a flat surface. This difference in the measured vs. expected EASA may be due to the coalescence between adjacent wrinkles, which hinders the solution from penetrating, as hypothesized previously for gold layers thinner than 50 nm [17]. Nevertheless, two sample t-test analyses (data not shown) proved that this is a statistically significant difference, with a size effect or Cohen’s d of 7.98. This might point out that the ANSEs should have a sensitivity per unit area at least 3 times larger than the arrays of flat-surface electrodes.

The EASA ratios between hook-shaped (counter) electrodes and straight (working) electrodes were 1.31 for flat-surface electrodes and 1.41 for structured-surface electrodes. Some references established that the counter-electrode EASA should be at least 10 times greater than the working electrode EASA; nevertheless, the measured difference proved to be sufficient for the array to function as an electrochemical cell handling currents of up to 500 µA. A two-sample t-test (two electrodes of each type evaluated two times, extracting three EASA data points from each experiment, n = 12, α = 0.05) showed that this was a significant difference, with effect size of 2.91 and 4.80 for flat-surface and structured-surface electrodes, respectively.

### 3.2. ANSE Characterization as Electrochemical Cells in the Diffusion-Controlled Mass Transport Regime

The performance of the arrays of nanowrinkled surface electrodes (ANSE) as electrochemical cells or sensors was evaluated using several standard methods. Cyclic voltammetry and chronoamperometry techniques were implemented only with the fabricated ANSE or in conjunction with a commercial Ag/AgCl reference electrode, using the ad hoc connection platform and the source meter unit working in potentiostat mode (Figure 2). We used the reversible redox pair ferrocyanide/ferricyanide ([Fe(CN)_6_]^3−^/[Fe(CN)_6_]^4−^) in 0.5 M potassium chloride as the standard system for electrochemical characterizations.

**Figure 4:**
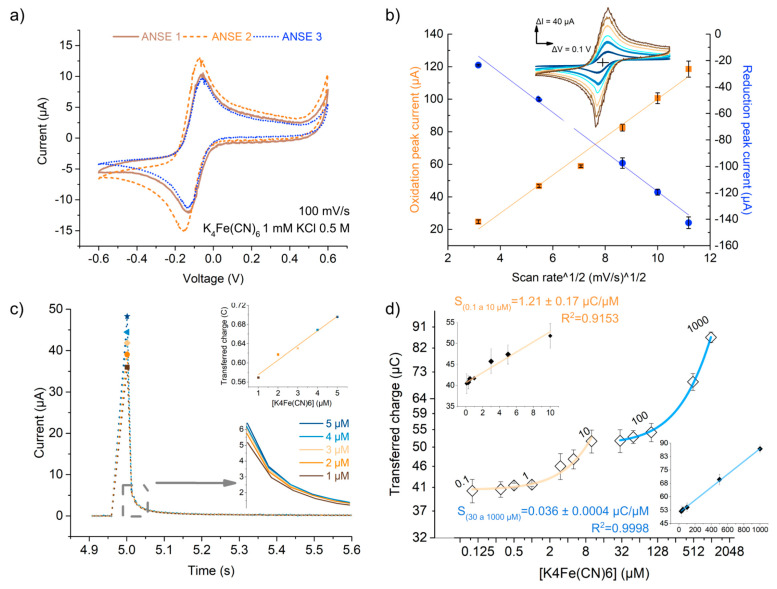
ANSE performed adequately as electrochemical cells when in the diffusion-controlled mass transport regime. (**a**) Cyclic voltammograms in 1 mM ferricyanide of different arrays of nanowrinkled-surface electrodes (ANSEs) showing good device-to-device reproducibility. (**b**) Fitting to the Randles–Sevcik model linking cyclic voltammetry scan rate and amplitude of oxidation and reduction peaks. (**c**) Chronoamperometry response of the ANSE to increasing concentrations of K_4_[Fe(CN)_6_] in the low 1 to 5 µM range; bottom inset shows a close-up of the enclosed section of the graph, and the top insert shows a plot of the Qtr response vs. concentration displaying a linear correlation. (**d**) Integrated transferred charge vs. K_4_[Fe(CN)_6_] concentration in a wide range shows good linearity but is concentration-dependent; axes are presented in log_2_ scale for visualization purposes. Data in (**b**,**d**) presented as mean ± standard deviation (SD); n ≥ 3 is the number of measurement repetitions using the same ANSE device.

The arrays alone functioned as electrochemical cells to perform cyclic voltammetry (C-V) sweeps. The characteristic duck shape of the reversible redox system [Fe(CN)_6_]^3−^/[Fe(CN)_6_]^4−^ was recovered without the aid of other external reference or counter electrodes. Good device-to-device reproducibility was observed, with coefficients of variation of 15% for the maximum and minimum currents and of 17% for the voltage at maximum and minimum current (Figure 4a). Differences may have arisen from reproducibility errors when defining the detection area, for this was only controlled via the position in which the PDMS well was manually adhered to the substrate with the electrodes (Figure 2b). Differences in the gold layer thickness may have also contributed to these errors.

In a system comprising electrochemically reversible electron transfer processes and freely diffusing electroactive species, when cyclic voltammetry sweeps are performed, faster voltage scan rates generate smaller diffusion layers and, as a consequence, higher faradaic currents. The Randles–Sevcik equation models the dependence of peak current *i_p_* (*A*) with the scan rate *v*:

(1) $$i_{p}=(2.68\times 10^{8})n^{3/2}AC_{o}\sqrt{Dv}$$

where *n* is the number of electrons transferred in the redox event, A (cm^2^) is the electrode surface area, D (cm^2^ s^−1^) is the diffusion coefficient of the oxidized analyte, and $C_{o}$ (mol cm^−3^) is the bulk concentration of the analyte [21,25]. The compliance of the current response of the ANSE device with the Randles–Sevcik equation was evaluated to gain insight into the mass transport and the electron transfer kinetics phenomena that govern the electrochemical measurements. Each point in the plot in Figure 4b displays the average oxidation or reduction peak current obtained from eight C-V scans carried out at the same scan speed with the same device in a solution containing 5 mM of K_4_[Fe(CN)_6_], 5 mM of K_3_[Fe(CN)_6_, and 0.5 M KCl. Linear correlation is observed between the peak currents and the square root of the scan speed, with an R^2^ value larger than 0.98 in both cases (oxidation and reduction) and a random distribution of residuals (Figure S2a and Table S1). This implies that the system complies with such a model; that redox species are freely diffusing rather than adsorbing in the gold surface despite its nanostructuring; and most importantly, that the system withstands reversibility even at scan speeds as high as 125 mV/s for this concentration range. Nevertheless, increasing separations of peak-to-peak voltage and current as a function of scan rate were observed (Table S1), possibly due to the use of a nonideal reference electrode. To investigate this further, the same experiment was repeated using a reference Ag/AgCl instead of the pseudoreference gold electrode of the array (Figure S2b). The expected linear correlation was verified for both the oxidation and reduction peak currents; nonetheless, a relation between the peak’s separation and the scan rate was not well defined.

Newly prepared electrodes were used for the chronoamperometric detection of ferrocyanide in the concentration range of 1 to 5 µM through its electro-oxidation at 0.5 V (against the pseudoreference electrode). We wanted to preliminarily assess whether the system could be used to detect nitric oxide released from cells in culture using the same electrochemical technique. This range was selected because it includes the estimated nitric oxide concentration after the excitation of endothelial cells [26,27]. A linear correlation between the peak current and the analyte concentration was detected, as predicted by the Cottrell equation for the step potential process with mass transport in the diffusion regime:

(2) $$i(t)=\frac{nFAD^{1/2}C_{o}}{\pi^{1/2}t^{1/2}}$$

where F is the Faraday constant; t is the time after the potential step application; and A,D, and $C_{o}$ are the same as in Equation (1).

The linear correlation was also verified for the transferred charge in a 0.5 s window (Figure 4c insets). The maximum current response for such small concentrations was up to five times higher than those registered at C-V scans for a 1 mM solution of K_4_[Fe(CN)_6_] This might be due to the presence of capacitive discharge currents occurring after the 0 to 0.5 Volts step change, which might be large for this type of electrode because of the larger EASA, implying a wider double layer and thus a larger capacitance. Figure S3a compares the plots of current as a function of time-derived scales, $t^{-1/2}$ or exp(−t). It is observed that the current fits better to a $t^{-1/2}$ decay, meaning it follows the Cottrell equation, instead of decaying as an inverse exponential function, as a capacitor discharge current does. This signifies that the registered current has a larger contribution from the faradaic process even if the maximum is increased because of discharge current contributions.

The response of the ANSE to ferrocyanide concentrations in the large range of 0.1 to 1000 µM was evaluated in order to further characterize the analytical parameters of the system. Despite the fact that the peak current did not grow linearly but rather asymptotically with concentration (Figure S4a,c), it can be derived from Cottrell’s (Equation (2)) equation integration that the amount of charges originating in the faradaic processes should be proportional to the analyte initial bulk concentration. We found that the transferred charge showed a range-dependent linear correlation with the concentration, as shown in Figure 4d (residuals plots shown in Figure S4b,d). From 0.1 to 10 µM, the device presented a sensitivity of 1.21 ± 0.17 µC/µM, while between 30 and 1000 µM, the fit slope was 0.036 ± 0.0004 µC/µM. This corresponds to sensitivities per unit area of 1.865 µC µM^−1^ cm^−2^ and 0.055 µC µM^−1^ cm^−2^, respectively. From blank (KCl 0.5 M) chronoamperometry measurements, two different sets of limit parameters were calculated, as shown in Table S2. It is also important to mention that the chronoamperometric measurement repetitions were performed with the same array of nanowrinkled surface electrodes, and the resulting transferred charge of each ferrocyanide concentration showed high repeatability, with 6.4% being the largest standard deviation (error bars in Figure 4d). This result is a sign that the ANSE device can be reused at least four times with high repeatability.

The same calibration experiments were performed with electrochemical cells including a commercial electrode option for comparison (Figure S5). On one side, we used the ANSE with the Ag/AgCl reference electrode (RRPEAGCL, PINE Research) instead of the pseudo-reference gold NSE. Peak current and transferred charge plots against ferrocyanide concentrations in Figure S5a,b show that this configuration also performs as a good electrochemical sensor but only in the concentration range of 30 to 1000 µM; at lower concentrations, linearity was not observed. The calculated sensitivity (0.011 ± 0.0008 µC/µM) was almost one third of the value obtained when using only the ANSE, which might be a consequence of a larger ohmic drop due to the longer distance between the reference and working electrode [28]. We also tested a commercial array of gold screen-printed electrodes (RRPE2021AU-6, PINE Research). Figure S5c,d show the corresponding plots of the obtained results. A linear correlation was not verified for the peak current or the transferred charge with the ferrocyanide concentration.

The electrochemical characterization results obtained here (and presented in the Supplementary Materials) confirmed that the fabricated arrays of nanowrinkled surface electrodes are suitable as electrochemical devices because they all required important properties for such sensors: high device-to-device reproducibility, good linearity, and high sensitivity for the detection of electroactive species in a concentration range suitable for the detection of biomarkers of interest in biological environments.

### 3.3. Detection of Nitric Oxide Released by Stimulated Endothelial Cells

The amperometric response of the ANSE towards nitric oxide (NO) concentrations was then studied with the aim of investigating the applicability of the device as an electrochemical sensor of biologically relevant molecules that are produced locally by endothelial cells under certain types of stimuli. It is of our particular interest to monitor this for cells cultured in traditional and microfluidic platforms, as a response to mechanical or chemical stimuli. Because it is more reproducible and controllable, we chose to employ a simple model of the endothelial cell synthesis of nitric oxide as a response to a specific concentration of a pro-angiogenic factor, the vascular endothelial growth factor (VEGF) [29], as a proof-of-concept application of the electrochemical sensor.

**Figure 5:**
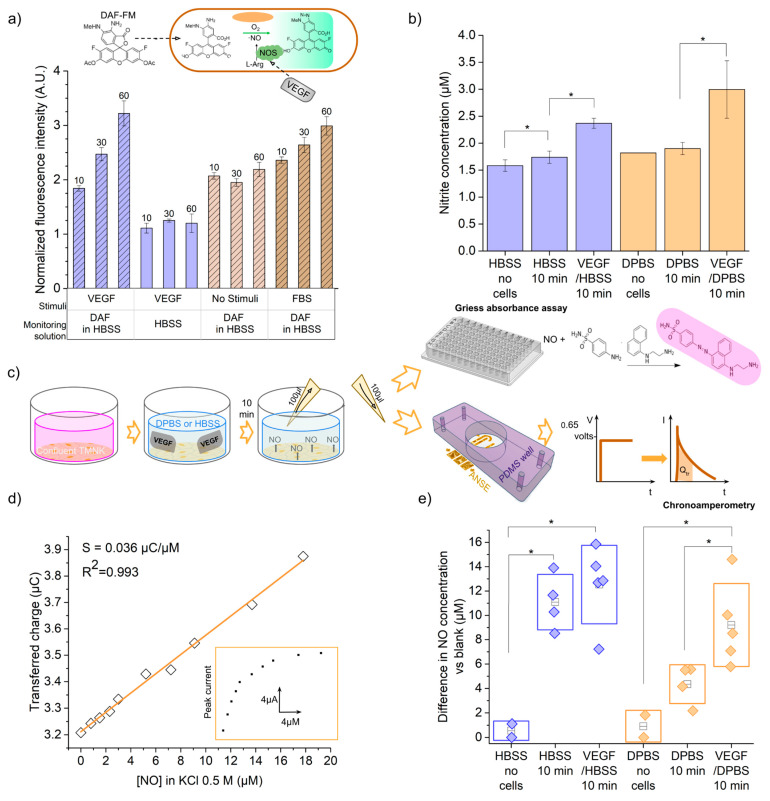
Detection of nitric oxide from endothelial cells extracellular media. (**a**) DAF-FM fluorescence assay results for intracellular NO detection. (**b**) Griess absorbance assay for solubilized NO detection. (**c**) Diagram of the process of stimulation of NO production in endothelial cells and subsequent detection with the absorbance Griess assay or the electrochemical sensor. (**d**) Calibration plot of ANSE’s transferred charge response vs. nitric oxide gas concentrations in a solution of KCl 0.5 M (supporting electrolyte). (**e**) Nitric oxide concentration (difference vs. blank) released by cells basally or after VEGF stimulation, as detected with the ANSE electrochemical sensor and calculated from the calibration curve. Statistical significance: * At *p* < 0.05 (n > 3).

In order to estimate the endothelial-released NO concentration, we measured one of its oxidation products, nitrite ions ${NO}_{2}^{-}$, in the extracellular media of stimulated endothelial cells by means of the standard colorimetric Griess assay. An absorbance vs. nitrite concentration curve was prepared for the 0.3 to 20 µM domain (Figure S6a), as indicated in the manufacturer’s protocol. From the linear fit, y = 0.00638x + 0.000387, and the absorbance obtained for each extracellular medium, the nitrite concentration was calculated. The average nitrite concentration obtained for each solution is shown in Figure 5b. As observed in the bar graph, the Hank’s balanced salt solution 1× (HBSS 1×) and Dulbecco’s phosphate-buffered saline 1× (DPBS 1×) solutions alone present absorbance signals that correspond to NO_2_^−^ concentrations of 1.58 µM and 1.82 µM, respectively. These results reveal the presence of nitrite ions as impurities in the buffer solutions, as ideally, no nitrite should be present with the absence of cells secreting NO. The concentration of nitrite in the HBSS and DPBS solutions that were in contact with the cells was higher, respectively, 1.74 and 1.9 µM, which corresponds to a difference of 0.16 and 0.08 uM, respectively. This accounts for a basal production of nitric oxide by endothelial cells, which was also confirmed after detecting nitrite in 24 h incubation media and new media incubated also for 10 min (Figure S6b). Finally, after 10 min of stimulation with VEGF, a greater average concentration of 2.37 µM was quantified in the solution with HBSS 1× background. This represents an increase of 0.63 µM compared with the basal production. In the case of VEGF in DPBS 1×, the measured concentration difference was 1.48 µM, when compared with the basal production. In both cases, the differences were calculated to be statistically significant under the two-sample *t*-test with a *p* < 0.05. We also verified that 10% FBS quickly triggers nitric oxide synthesis, as shown in Figure S6b, since it contains various stimulant factors [30]. Additionally, a cell viability test, in the form of a calcein AM/PI stain assay, was performed and showed that cells retained their viability after the 10 min incubation with the stimulant (Figure S6c).

To further confirm that the Griess assay was measuring a nitric oxide signal from endothelial cells, we performed an additional optical detection of the intracellular nitric oxide production, based on the fluorescence of diaminofluorescein FM diacetate (DAF-FM diacetate). This molecule is internalized by the cells and transformed into DAF-FM via the action of intracellular esterases. Once it reacts with the nitric oxide synthesized by the enzyme eNOS (endothelial nitric oxide synthase), in the presence of oxygen, the triazolo-fluorescein analogue (DAF-FM T) is formed (Figure 5a, inset). DAF-FM T has an excitation wavelength of 500 nm, an emission of 515 nm, and a quantum efficiency 160 times greater than the initial molecule, which can be detected in an epifluorescence microscope as an increase in fluorescence intensity (see Figure S7 for micrographs). It is not possible to quantify the concentration in the extracellular medium from such detection, since the molecule needs to be internalized into the cells. Nevertheless, it is a specific assay for intracellular nitric oxide, which can provide information in real time.

The average fluorescence intensity from the cell layers at different stimulation and capture time conditions was normalized against the smallest gray value measured, and the results are plotted in Figure 5a. For the layer of VEGF-stimulated cells immersed in only HBSS (no DAF-FM), a very small basal value of fluorescence intensity was detected, corresponding to the possible remaining GFP expression from TMNK-1 cells [31]. The fluorescence of VEGF-stimulated cells immersed in HBSS plus DAF-FM was 2.7 times larger than the basal fluorescence. This verifies that the fluorescence is due to the DAF-FM molecule and not to the GFP expressed by the cells. On the other hand, when the cells were cultured in a medium without stimulation (serum and VEGF-free media), the addition of the DAF-FM molecule still produced a fluorescent signal. This means that the cells indeed produce a basal amount of nitric oxide, as also detected with the previous Griess assay; however, no significant accumulation of nitric oxide was detected after 10 min. Finally, it was observed that cells stimulated for two hours with the growth factor or cultured in fetal bovine serum maintain nitric oxide production for up to 60 min. In cells stimulated with VEGF, after 60 min, nitric oxide reached a level 1.75 times higher than the value measured at 10 min; furthermore, at only 10 min, they have a NO level that is 1.19 times higher than the basal value of cells that were not stimulated (cells with only DMEM without FBS).

Despite the fact that the NO concentrations detected in the extracellular medium were in the lower portion of the amperometric response calibration curve, between 1.5 and 2.4 µM (compare Figure 4d and Figure 5b), it was expected that the electrochemical sensor would be capable of detecting these differences as it did for the oxidation of ferrocyanide in concentrations from 0.1 to 10 µM, with high sensitivity.

Chronoamperometric measurements to solutions of increasing NO concentration were performed with an ANSE to obtain the response curve. During our first attempts, we tested using DPBS 1× as the supporting electrolyte because we expected to integrate the sensor with the culture and stimulation device. We did not succeed in obtaining the typical linear response of either the current or transferred charge increasing with the analyte concentration (Figure S8a,b). We repeated the test for the chronoamperometric detection of ferrocyanide using DPBS 1× as a supporting electrolyte, but again, no correlation was observed.

Considering this, we decided to perform the calibration and detection experiments in 0.5 M KCl, as usual. The nitric oxide concentration of the first 1:20 dilution from the stock solution, as quantified using the Griess assay, resulted in 79 µM. An amount of 100 µL of a 0.5 M KCl solution was poured in the PDMS well adhered to the sensor; then, 5 µL aliquots of the 79 µM NO solution were injected into the initial volume with a Hamilton syringe and the 0.65 V potential step was implemented right after each addition. The transferred charge for each current peak response was plotted against the final NO concentration (from 0.8 to 17.8 µM), as shown in Figure 5d. A positive linear relationship was verified (Figure S8c), and the least squares regression yielded the equation $Q_{tr}=0.036*[NO]+3.21$, where $Q_{tr}$ is the transferred charge in µC and [NO] is the nitric oxide concentration in µM. The slope, 0.036 ± 0.0009 µC/µM, represents the sensitivity of the sensor towards nitric oxide concentration. This is congruent with the sensitivity obtained from the ferrocyanide electrooxidation with the same device configuration. The sensitivity per unit area, analogously, is 0.055 µC µM^−1^ cm^−2^. More analytical parameters can be found in Figure S8d.

Finally, we carried out an experiment to conduct the electrochemical measurement of nitric oxide in the extracellular media of the stimulated cells in real cell culture conditions (Figure 5e). We implemented the same culture and stimulation parameters used for the Griess assay. A total of 20 µL of a 3 M potassium chloride solution was placed in the PDMS well on top of the array of electrodes; then, 100 µL of the extracellular solution to be analyzed was added. Chronoamperometry was implemented with the same parameters as for the calibration curve, and the transferred charge at each current peak was calculated in a 1 s window. The nitric oxide concentration in the solution was calculated using the calibration curve $Q_{tr}([NO])$. To account for only the nitric oxide produced by the cells during the 10 min incubation, we subtracted the corresponding signal from the background solutions that had no contact with the cells. These values are plotted in Figure 5e, and the significant differences under the two-sample *t*-test are indicated. The average nitric oxide concentration detected in the stimulation media surrounding the endothelial cells was 1.13 times larger than the concentration found for the unstimulated cells in the case of HBSS, whilst for DPBS, it was 2.11 times larger. This proved that our sensor is capable of monitoring a NO secretion of endothelial cells as a response to a VEGF stimulus under normal culture conditions.

## 4. Discussion

In this work, we have designed, fabricated, and successfully tested devices consisting of arrays of nanowrinkled surface electrodes (ANSE) on plastic substrates for the detection of NO production by endothelial cells cultured in vitro. We have presented the benchtop fabrication procedure and characterization results exhaustively to help readers reproduce these promising results in their own laboratory, with a future aim to integrate such microelectrodes inside a microfluidic device for reliable, real-time, in situ measurements. Previous work has reported the fabrication of similar nanostructured surface electrodes [15,16,17,18,19], but in this work, the fabrication technique was extended to develop fully functional planar arrays of electrodes which serve as electrochemical cells for amperometric techniques where the species mass transport is controlled by diffusion. Other groups have reported on the fabrication of integrated electrochemical sensors before but they used alternative reference electrodes, like a salt-bridge-free structured platinum electrode [10], or they explored the functionality of the arrays transferred to flexible substrates and under voltammetric techniques [32,33].

We demonstrated that only a 30 nm gold deposition thickness is necessary for functionality, compared with the 100 or 200 nm thickness used in previous reports [18]. Increasing the gold film thickness, though, might be useful to decrease the coalescence between adjacent wrinkles and thus to increase the EASA, as measurements from Gabardo et al. have shown [17]; unfortunately, this was not possible with our setup because longer sputtering times increased the chamber temperature and triggered the PS shrinkage. We also recommend, as implemented here, cutting the mask after the tape adheres to the substrate because it prevents the gold film from tearing after mask detachment thanks to the trench-like relief observed in the inset image in Figure 3bi. Moreover, this reduces the number of fabrication steps and guarantees better reproducibility.

To our knowledge, this is the first time that an extensive characterization of the electrochemical performance of these three-electrode devices is presented, proving their use as sensitive electrochemical amperometric sensors in the low micromolar, or even submicromolar, range, displaying good linearity and limit of detection. Furthermore, it was verified that this simple design is suitable for use in the detection of nitric oxide in concentration ranges as low as the micromolar range, as long as the appropriate supporting electrolyte is used. Specifically, we obtained a sensitivity value of 0.036 ± 0.0009 µC/µM, or 0.055 µC µM^−1^ cm^−2^ if talking about sensitivity per unit area. Considering that we make a 1 s integration from the maximum current, we could estimate that our sensor’s sensitivity would take a maximum value of 0.036 µA/µM, which is in the upper portion of the reported range of electrochemical sensor’s sensitivities (0.00018 to 0.123 µA/µM), summarized in the excellent review by Brown et al. [26], despite our sensor not needing a chemical modification for the selective detection or catalytic oxidation of NO.

The electrochemical sensor literature reports the electrochemical detection of nitric oxide while using PBS or HBSS as supporting electrolytes, while the cultured cells in the vicinity are stimulated chemically [11,34,35,36]. Very few, though, report on the calibration of the electrochemical response to different NO concentrations under the same electrolyte conditions; even reports of ferrocyanide electro-oxidation using a PBS as the supporting electrolyte are sometimes unclear [37]. Some carbon-fiber- or glassy-carbon-based devices have been used for NO electrooxidation calibration experiments, but they use alternative techniques such as differential pulse or square-wave voltammetry [38]. We chose chronoamperometry due to its high sensitivity and the simplicity of the instrument needed and because it is always included in low-cost potentiostats scripts. Nevertheless, we were only able to obtain the ideal response when the common supporting electrolyte, potassium chloride KCl, was used. This is a very important point identified in our work and is usually not highlighted in similar assays.

The need for KCl as a supporting electrolyte may limit the use of this device for repeated in situ measurements because the osmolarity mismatch would kill the cells, but some simple adaptations, like the integration of additional channels and pumps, would make it possible to transport the extracellular medium to the sensing area, where KCl could be incorporated in concentrations greater than 0.5 M without affecting cell viability and without the need for heavy repeated pipetting for well plates.

We also conclude from the comparison between using only the ANSE or adding the Ag/AgCl reference electrode that integrating all three electrodes in a planar array is very advantageous. Indeed, the working and reference electrodes are closer together so the solution resistance between them is smaller, thus giving a lower potential ohmic drop. On the other hand, the low analyte concentrations produce small faradaic electric currents between the electrodes, which are not sufficient to yield a significant reference potential change; thus, it is not necessary to include a real reference system for this concentration range [39].

Finally, with our system, it was possible to rapidly measure endothelial cell secretion of nitric oxide in concentrations lower than 2.4 µM, as a response to only 10 min of interaction with a VEGF stimulation at a common concentration of 50 ng/mL. After applying chronoamperometry with the ANSE device, a higher transferred charge was observed in the measurement of the stimulation extracellular media. The comparison of unstimulated and stimulated cells was also consistent for both the chronoamperometric and the absorbance Griess assays, but the chronoamperometric measurements implied a much shorter processing time. However, the electrochemical detection might be overestimating the nitric oxide concentration, as observed when comparing the electrochemical and the absorbance measurements (Figure 5b,e). This may be caused by the electrochemical detection of other reactive species, i.e., other molecules that the cells have released into the supernatant extracellular medium may be oxidizing at this overpotential and then contributing to the total transferred charge measured by our device, accounting for incomplete specificity. This selectivity problem could be solved by adding coatings such as Nafion, which prevents the passage of negatively charged molecules, such as ascorbate, nitrite, or nitrate ions, and allow, due to its pore size, the passage of small molecules [40,41]. However, the proper and efficient bonding of Nafion to the nanowrinkled or nanostructured gold electrodes has not been demonstrated yet and would require more work to prove its suitability [42]. Another alternative would be to immobilize, on the surface of the working electrode, some molecules that selectively form a complex with NO, such as porphyrins, metal phthalocyanine complexes, and its derivatives [35,43]; this would ensure that only NO molecules are in the vicinity of the electrode surface and thus that the anodic current is due to its oxidation and not to other interfering molecules.

Overall, we believe that the presented arrays of nanowrinkled surface electrodes (ANSE) are suitable as electrochemical sensors to detect electroactive analytes in the wide submicromolar to submillimolar concentration range, in addition to having low fabrication and instrumentation costs, high sensibility, and good reusability. Since we were able to detect NO production by endothelial cells cultured in vitro, after simple modifications, we expect to use these devices for the in situ, real time, on-demand monitoring of biomarkers secreted by cell cultures in traditional or microfluidic biomimetic platforms in order to help resolve unanswered biological questions.

## Data Availability

All data relevant to the study are included in the article or uploaded as Supplementary Materials. They are available upon reasonable request to the authors.

## Supplementary Materials

The following supporting information can be downloaded at https://www.mdpi.com/article/10.3390/bios13080794/s1, Figure S1: Measurements of geometric dimensions of the fabricated electrodes; Figure S2: Further results for the Randless–Sevcik model validation of our ANSE device; Table S1: Summary of comparison results for the evaluation of the Randles–Sevcik model; Figure S3: Current decay as a function of different time-derived scales to define the best-fitting behavior; Figure S4: Chronoamperometric test response of the ANSE to increasing concentrations of ferrocyanide; Table S2: Summary of analytical parameters for the ANSE as an electrochemical sensor of ferrocyanide; Figure S5: Chronoamperometry responses of commercial electrodes; Figure S6: Nitric oxide detection with a Griess absorbance assay; Figure S7: Fluorescence images of DAF-FM after nitric oxide detection at the different stimulation or control conditions; Figure S8: ANSE’s response to nitric oxide electro-oxidation through chronoamperometry when using either DPBS 1× or KCl 0.5 M as supporting electrolyte.
