# Supplementary material for: Characterization of Benchtop-Fabricated Arrays of Nanowrinkled Surface Electrodes as a Nitric Oxide Electrochemical Sensor
Source: Biosensors (Basel). 2023 Aug 7;13(8):794. doi: 10.3390/bios13080794 (PMC10452632; doi:10.3390/bios13080794)
Supplement: Supplementary file 1 [file biosensors-13-00794-s001.zip › biosensors-2509664-supplementary.pdf]

biosensors

MDPI

Supplementary materials

Characterization of Benchtop-Fabricated Arrays of Nanowrinkled Surface Electrodes as a Nitric Oxide Electrochemical Sensor

Cindy Peto-Gutiérrez [1,2,*], Genaro Vázquez Victorio [1,2] and Mathieu Hautefeuille [1,3,*]

[1] Laboratorio Nacional de Soluciones Biomiméticas para Diagnóstico y Terapia (LaNSBioDyT), Universidad Nacional Autónoma de México, Mexico City 04510, Mexico; genvazquez@ciencias.unam.mx
[2] Departamento de Física, Facultad de Ciencias, Universidad Nacional Autónoma de México, Mexico City 04510, Mexico
[3] Laboratoire de Biologie du Développement (UMR 7622), Institut de Biologie Paris Seine, Sorbonne Université, 75006 Paris, France
* Correspondence: cinviry@gmail.com (C.P.-G.); mathieu.hautefeuille@sorbonne-universite.fr (M.H.)

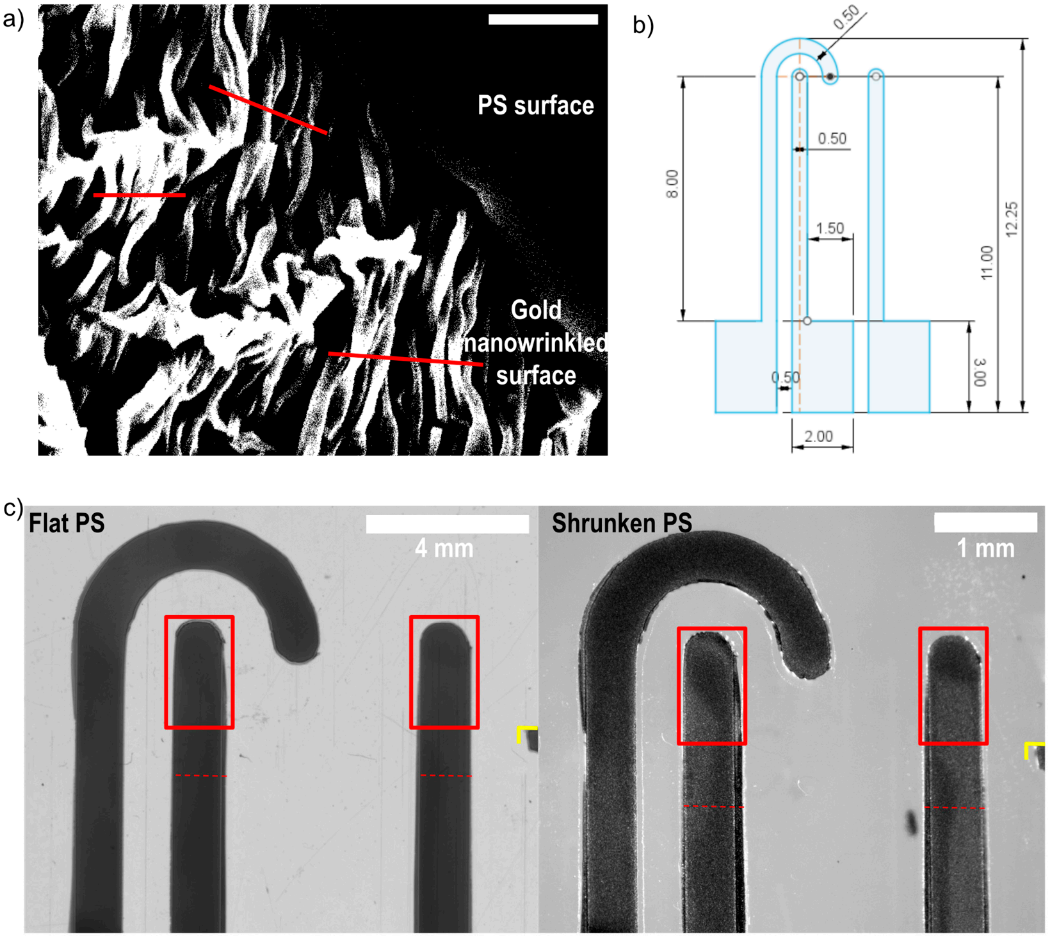

Figure S1. Measurements of geometric dimensions of the fabricated electrodes. (**a**) Transformed SEM micrograph of nanowrinkled gold surface to highlight the wrinkles width (same FOV as inset in Figure 3b), scale bar = 5 µm, some of the width measurement directions are indicated with red lines. (**b**) Design of the array of electrodes and the corresponding characteristic dimensions in mm. (**c**) Transillumination pictures of an array of electrodes before (left, bar = 4 mm) and after (right, bar = 1 mm) shrinking; red squares indicate the zone where the area measurements were performed to calculate the shrinking ratio. To measure the areas with ImageJ software, image was transformed to

32 bits, “Adjust threshold” was applied, the rectangular area was selected, aligned to the reference mark (yellow) and finally the “Measure” command yielded the “dark” area, corresponding to the gold surface.

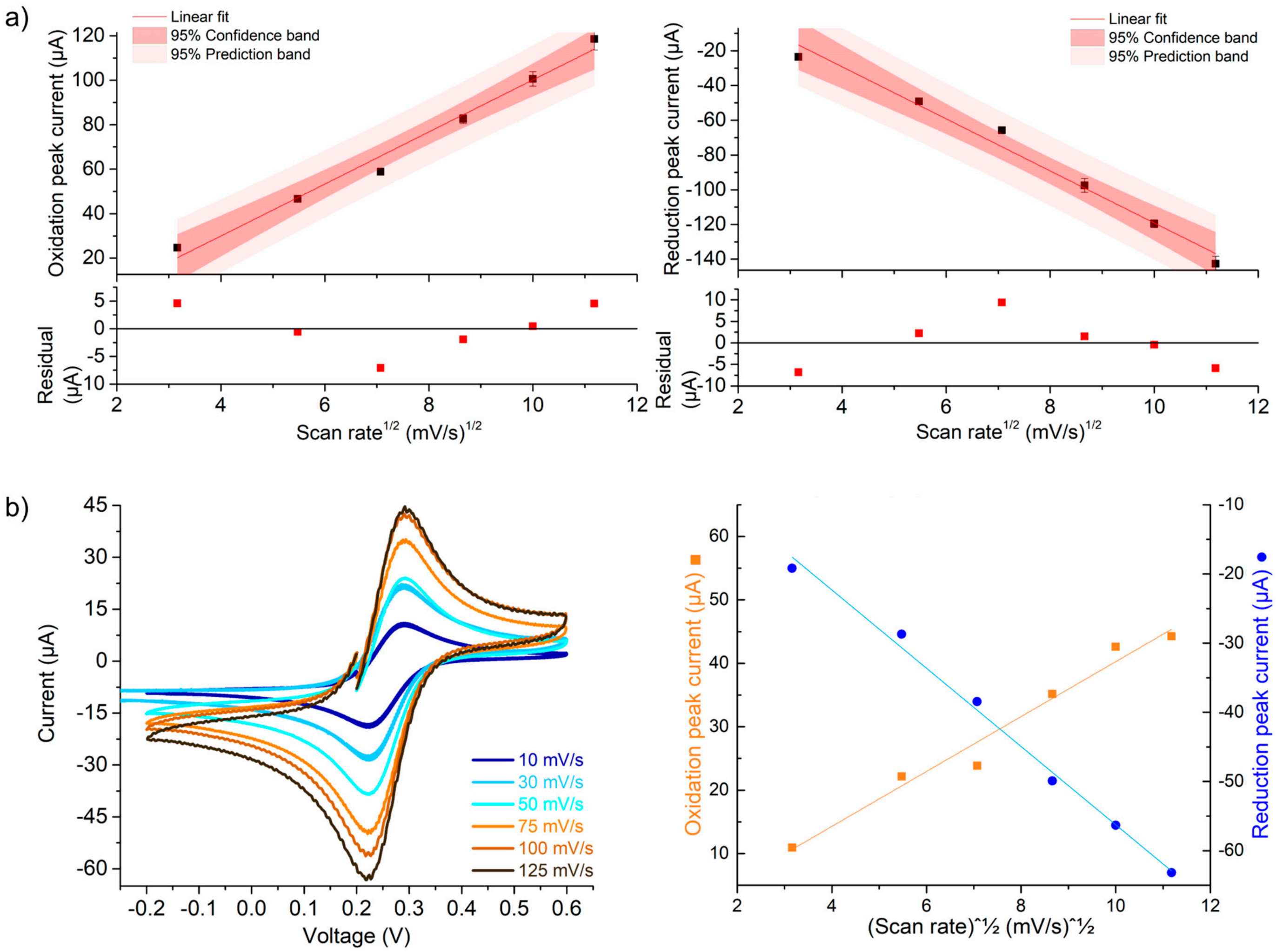

Figure S2. Further results for the Randless-Sevcik model validation of our ANSE device. (**a**) Plots of reduction (left) and oxidation (right) peak currents against the square root of the scan rate also showing the confidence and prediction bands and the residuals plot below. (**b**) Results of the Randles-Sevcik experiment performed with the ANSE and a real Ag/AgCl reference electrode; left: voltammograms displaying the current amplitude increasing as the sweep scan rate grows; right: plot of peak currents as a function of the square root of the scan rate and the corresponding linear fits.

Table S1. Summary of comparison results for the evaluation of the Randles-Sevcik model.

	**1-ANSE**	**2-ANSE + Ag/AgCl**
**Oxidation peak current linear regression**	$\boldsymbol{I}_{ox} = (11.2 \pm 0.66\ \mu A \cdot s^{½} \cdot mV^{-½})\ \boldsymbol{v}^{½} + (-14.6 \pm 5.32\ \mu A)$	$\boldsymbol{I}_{ox} = (4.3 \pm 0.35\ \mu A \cdot s^{½} \cdot mV^{-½})\ \boldsymbol{v}^{½} + (-3.0 \pm 2.93\ \mu A)$
**Reduction peak current linear regression**	$\boldsymbol{I}_{red} = (-14.5 \pm 0.87\ \mu A \cdot s^{½} \cdot mV^{-½})\ \boldsymbol{v}^{½} + (28.9 \pm 7.02\ \mu A)$	$\boldsymbol{I}_{red} = (-5.6 \pm 0.23\ \mu A \cdot s^{½} \cdot mV^{-½})\ \boldsymbol{v}^{½} + (0.33 \pm 1.88\ \mu A)$
**Average difference between oxidation and reduction peak voltages for all scan rates**	61.0 ± 4.91 mV	69.6 ± 4.58 mV
**Difference between oxidation and reduction peak heights as a function of scan rate**	Increases with v<br>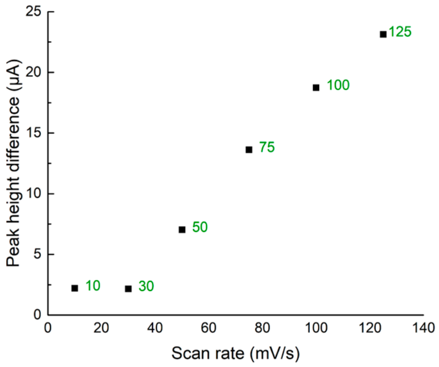	Unclear trend<br>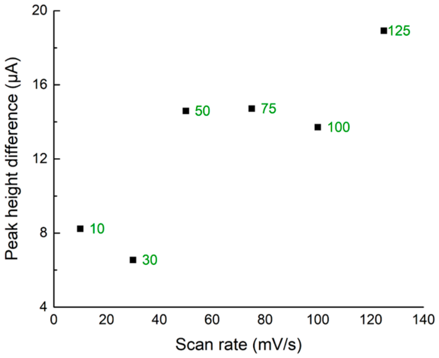

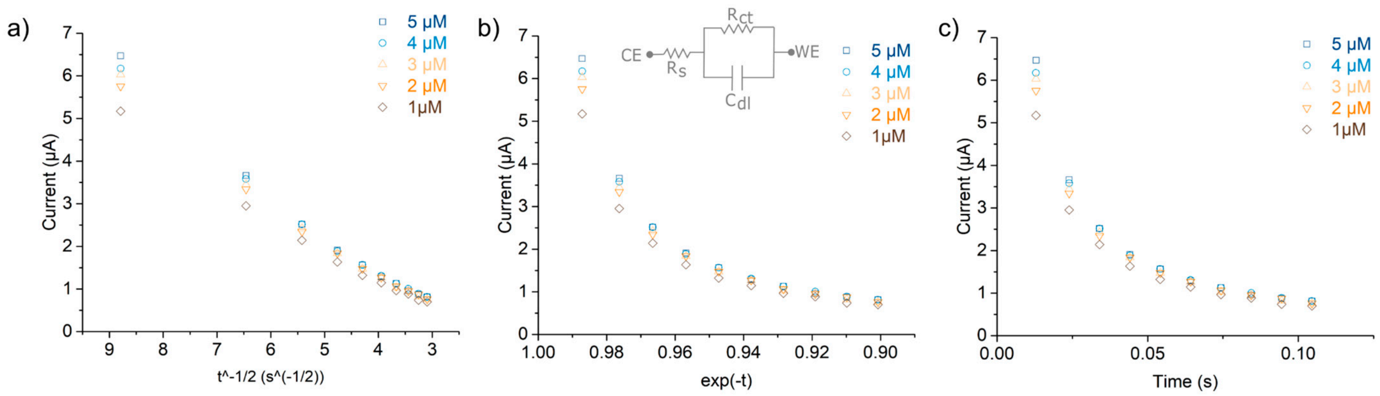

Figure S3. Current decay as a function of different time-derived scales to define the best fitting behavior; (**a**) as a function of $t^{-1/2}$ as predicted by Cottrell's equation for diffusion-controlled process; (**b**) as a function of $exp(-t)$, as in the discharge current decay (inset shows the typical equivalent circuit that is proposed to model the electric behaviour of the solution-electrode surface interface: Rs = solution resistance, Rct = charge transfer resistance - due to faradaic process, Cdl = double layer capacitance); (**c**) as a function of t.

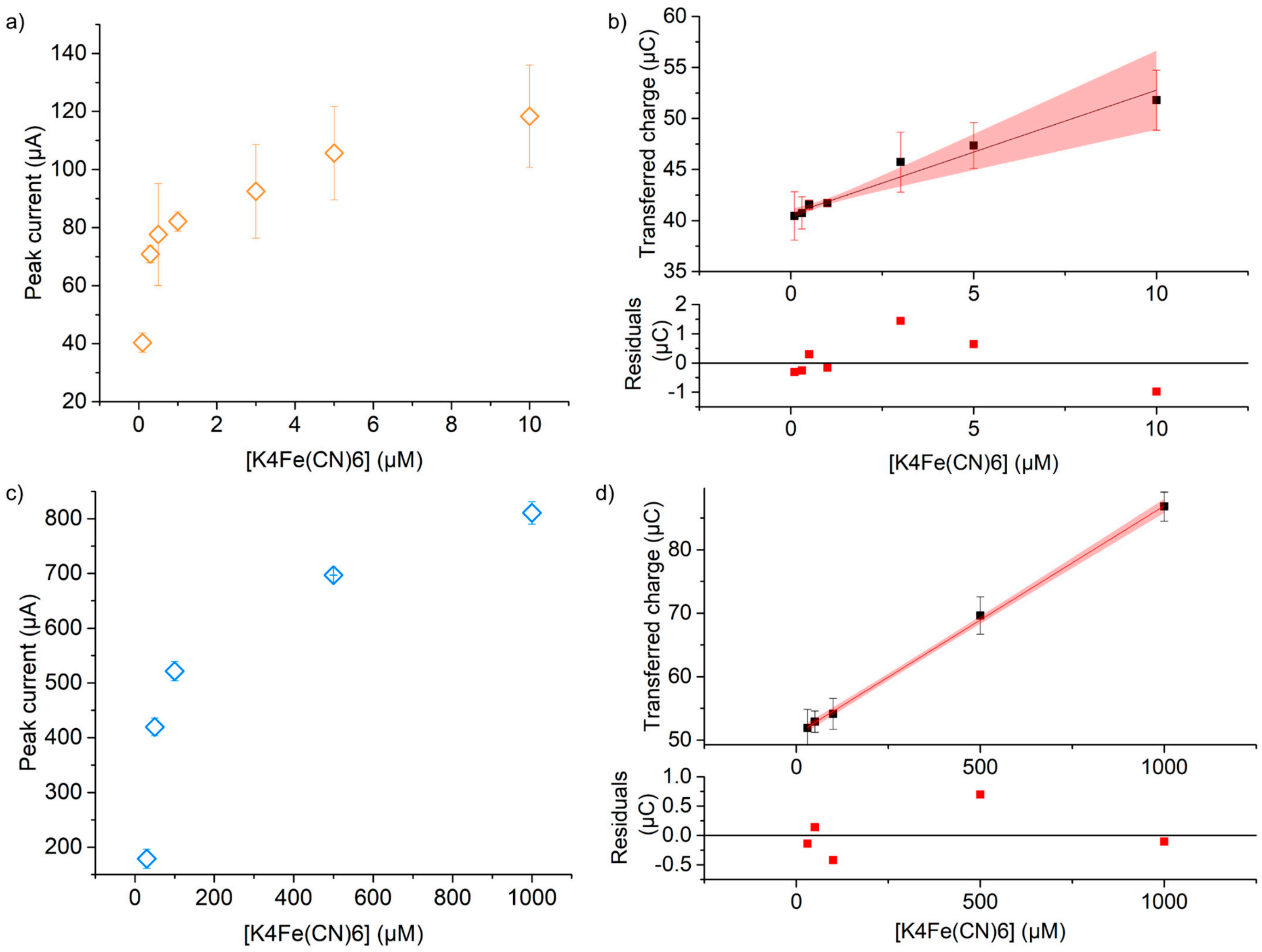

Figure S4. Chronoamperometric test response of the ANSE to increasing concentrations of ferrocyanide. (**a**) Peak current response as a function of ferrocyanide concentrations from 0.1 to 10 µM. (**b**) Transferred charge calibration curve, confidence interval and residuals plot for the 0.1 to 10 µM range. (**c**) Peak current response as a function of ferrocyanide concentrations from 30 to 1000 µM. (**d**) Transferred charge calibration curve, confidence interval and residuals plot for the 30 to 1000

µM range. Data presented as mean ± standard deviation (SD), n ≥ 3 is the number of measurement repetitions using the same ANSE device.

Table S2. Summary of analytical parameters for the ANSE as an electrochemical sensor of ferrocyanide.

**Sensitivity (Concentration Range-Dependent)**	**Fit from 0.1 to 10 µM:** $\mathbf{1.21 \pm 0.17\ \mu C/\mu M}$	**Fit from 30 to 1000 µM:** $\mathbf{0.036 \pm 0.0004\ \mu C/\mu M}$
**LOD = 3*SD/m** [µM]	0.827	27.910
**LOL** [µM]	10	1000
**LOQ = 10*SD** [µM]	2.758	93.033
**Dynamic range**	2.76 to 10 µM	93 to 1000 µM

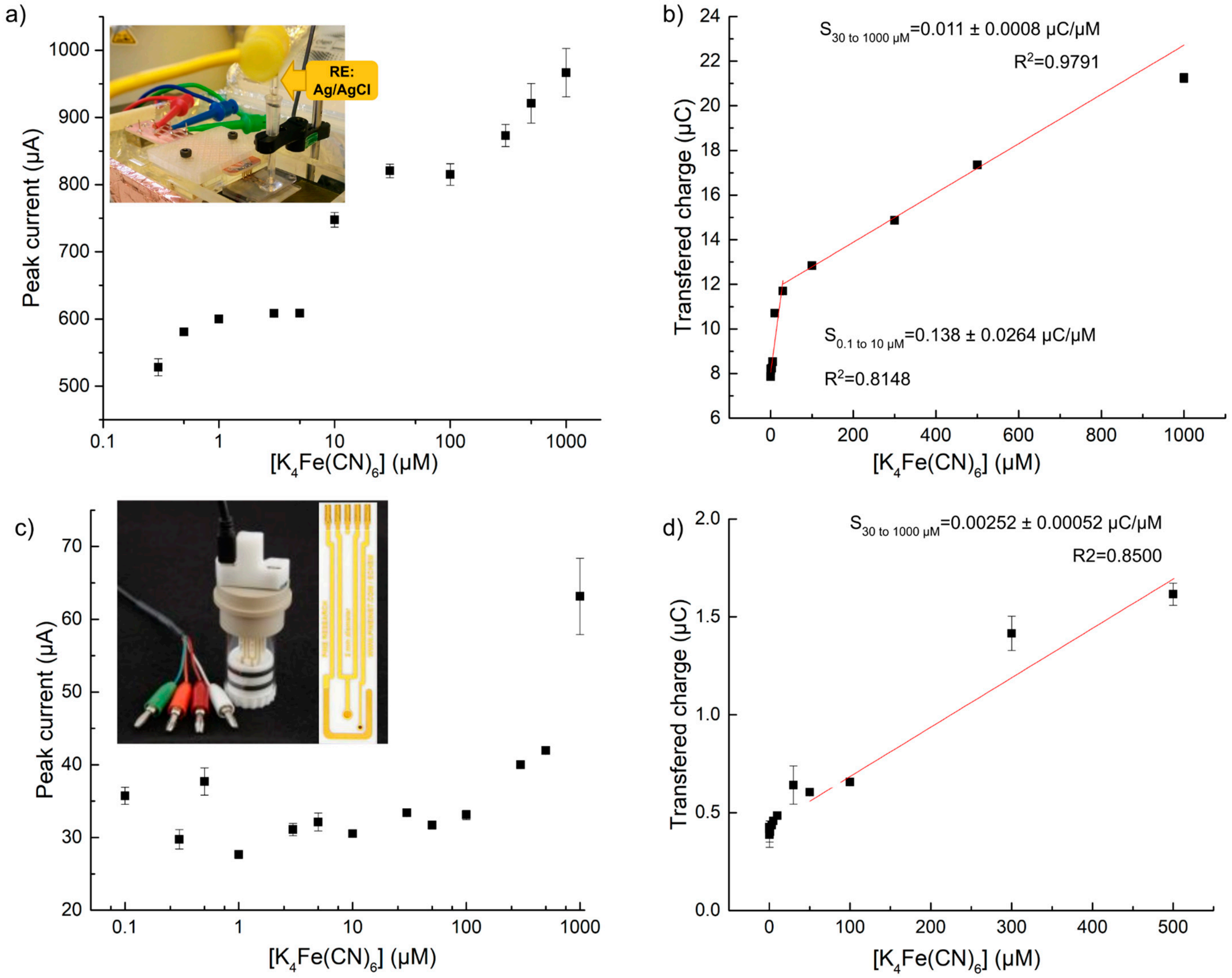

Figure S5. Chronoamperometry responses of commercial electrodes; (**a**,**b**) correspond to measurements using a cell consisting of a working and a counter NSE and a Ag/AgCl reference electrode; (**c**,**d**) correspond to the use of a commercial array of gold screen-printed-on-ceramic electrodes (cSPE). (**a**) Peak current response as a function of ferrocyanide concentrations (0.1 to 1000 µM). (**b**) Integrated transferred charge for each peak of each ferrocyanide concentration. (**c**) Peak current response of the commercial cSPE. (**d**) Transferred charge vs. ferrocyanide concentration for cSPE. Data

presented as mean ± standard deviation (SD), n ≥ 3 is the number of measurement repetitions using the same ANSE device.

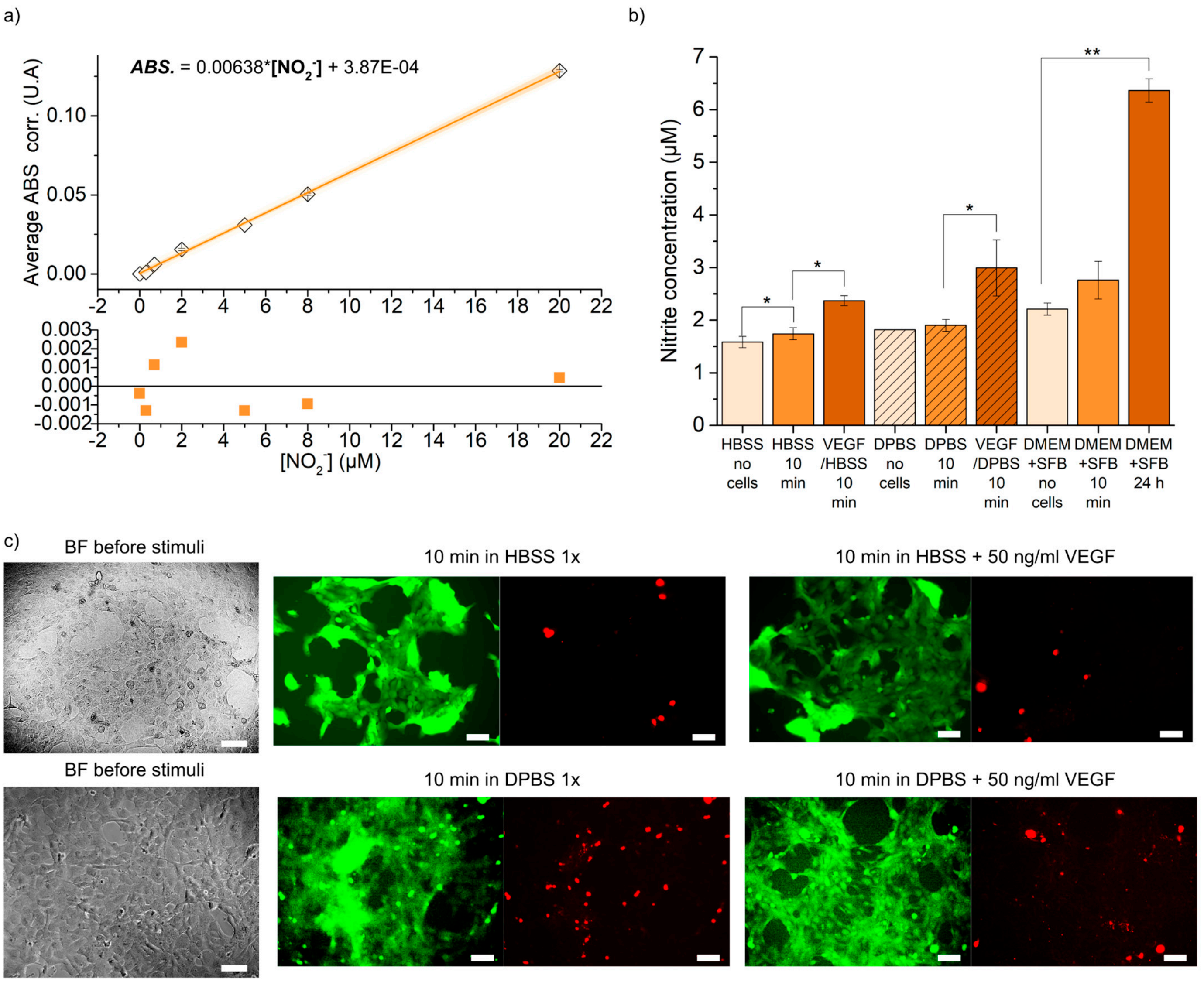

Figure S6. Nitric oxide detection with a Griess absorbance assay. (**a**) Calibration curve plot of corrected absorbance (subtracting blank result) vs. nitrite standard concentration, and the corresponding residuals plot. (**b**) Nitrite concentration detected in different extracellular media, the first 6 bars are the same as plotted in figure 5, we show for comparison the bars corresponding with the NO_2^- concentration detected in culture media (DMEM+SFB) alone, incubated for 10 min or for 24 h; statistical significance: **—at $p < 0.01$, *—at $p < 0.05$ (n > 3). (**c**) Results of viability assay after 10 min of incubation with buffer and VEGF stimulation solutions.

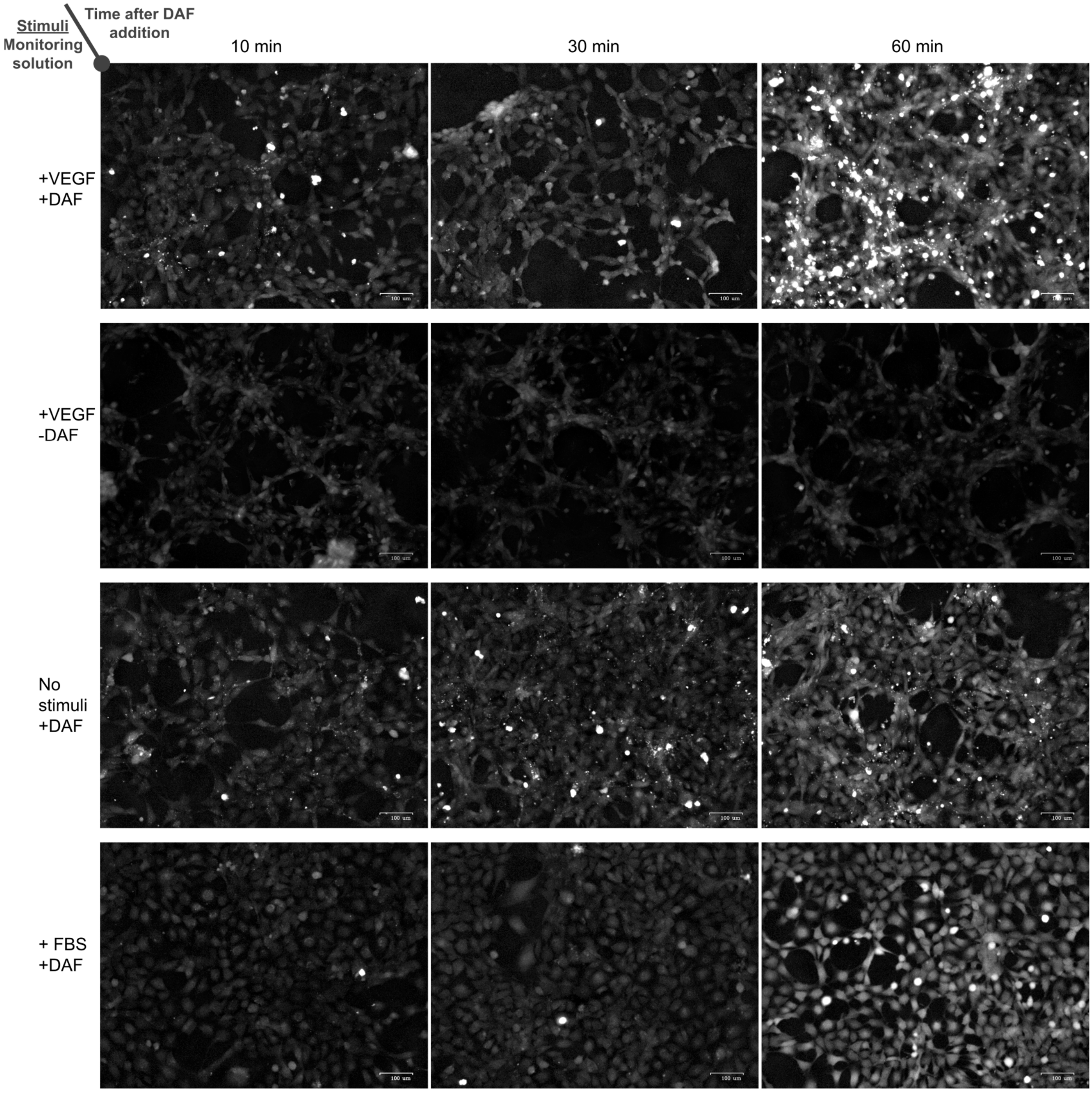

Figure S7. Images of the fluorescence of DAF-FM after reacting with intracellular nitric oxide at the different cells stimulation or control conditions and at different monitoring solution conditions (row names); three different observation times after adding DAF in HBSS or just HBSS to the living cells are also displayed (column names). Nomenclature: "+VEGF" = stimulation with VEGF 50 ng/mL, "+ FBS"= stimulation with 10% fetal bovine serum; + DAF or - DAF refers to the use of the monitoring solution, HBSS, with or without the fluorescent molecule, respectively.

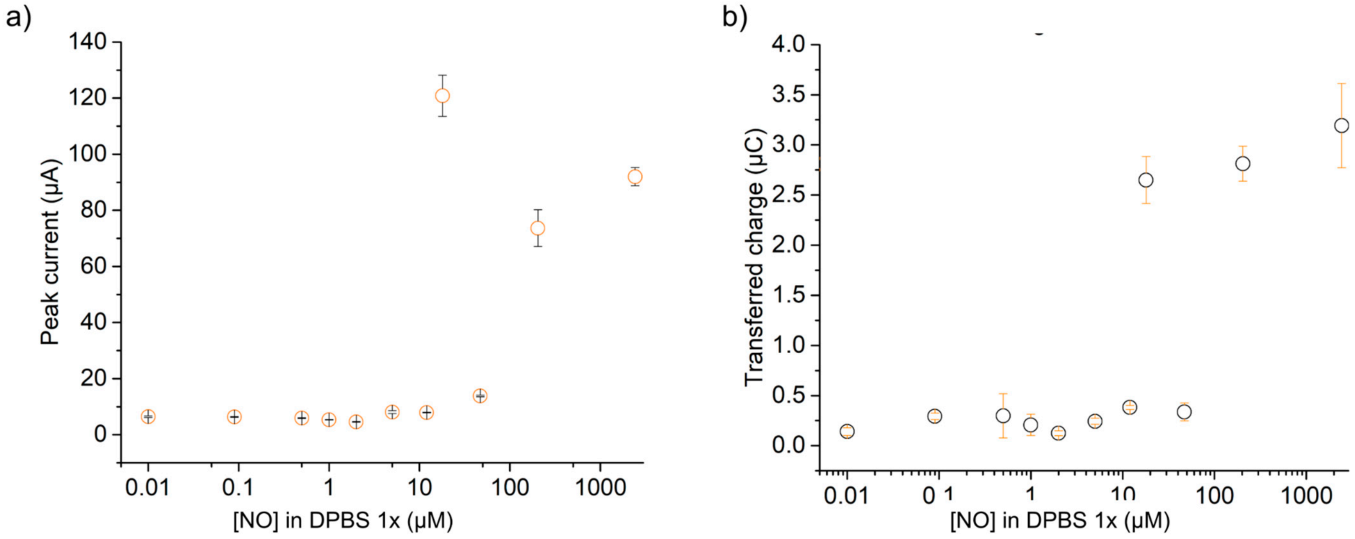

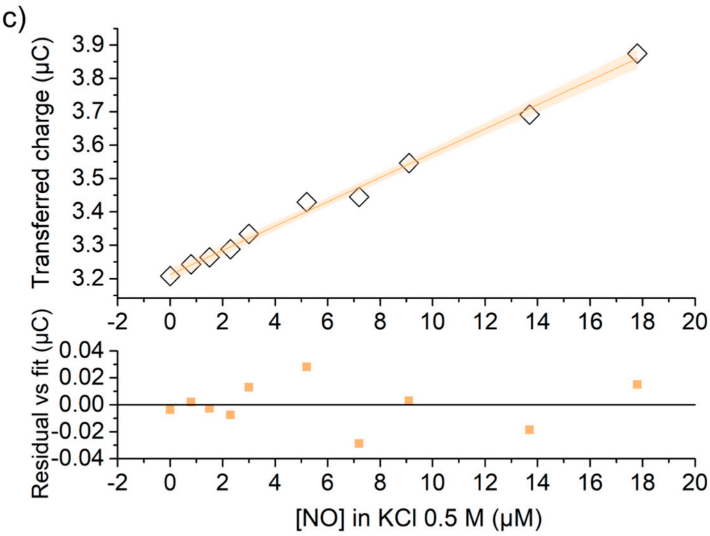

d)

**Sensitivity per unit area [µC µM^{-1} cm^{-2}]**
0.056
**LOD = 3*SD/m [µM]**
4.427
**LOQ = 10*SD/m [µM]**
14.758
**Dynamic range**
4.43 to 20 µM

Figure S8. ANSE's response to nitric oxide electro-oxidation through chronoamperometry when using either DPBS 1x (**a**,**b**) or KCl 0.5 M (**c**,**d**) as supporting electrolyte. Data in (**a**) and (**b**) presented as mean ± standard deviation (SD), n ≥ 3 is the number of measurement repetitions using the same ANSE device.
